# Tooth Loss Suppresses Hippocampal Neurogenesis and Leads to Cognitive Dysfunction in Juvenile Sprague–Dawley Rats

**DOI:** 10.3389/fnins.2022.839622

**Published:** 2022-04-28

**Authors:** Jiangqi Hu, Xiaoyu Wang, Wei Kong, Qingsong Jiang

**Affiliations:** ^1^Beijing Stomatological Hospital, Capital Medical University, Beijing, China; ^2^Key Laboratory of Molecular Medicine and Biotherapy, Department of Biology, School of Life Sciences, Beijing Institute of Technology, Beijing, China

**Keywords:** tooth loss, cognitive dysfunction, Alzheimer’s disease (AD), hippocampus, brain-derived neurotrophic factor (BDNF)

## Abstract

**Background:**

Both animal studies and prospective observational studies on patients with neurodegenerative disease have reported a positive link between oral diseases and cognitive function. However, the effect of early tooth loss on hippocampal morphology remains unknown.

**Methods:**

In this study, 6-week-old, male, juvenile Sprague–Dawley (SD) rats were randomized into the control (C) and tooth loss (TL) groups. In the TL group, all right maxillary molars of SD rats were extracted, while in the C group, no teeth were extracted. After 3 months, the learning and memory behavior were examined by Morris Water Maze (MWM), and the protein expression and mechanic signaling pathways were analyzed by real-time polymerase chain reaction, and cresyl violet staining.

**Results:**

Two days after the operation, the body weight of both groups recovered and gradually returned to the level before operation. Three months after tooth extraction, the completion time of the C group in the MWM was significantly shorter than the TL group. The mRNA expression of *BDNF, TrkB, AKT1*, and *NR2B* in the C group were significantly higher than in the TL group. The pyramidal neurons in the TL group was fewer than in the C group.

**Conclusion:**

Tooth loss in the juvenile SD rats will reduce the number of pyramidal neurons in the hippocampus, inhibit the expression of *BDNF, TrkB, AKT1*, and *NR2B*, and eventually lead to cognitive dysfunction.

## Introduction

The population of aging people in the world has considerably increased, which is related to an increasing number of Alzheimer’s disease (AD) cases in the world. According to research, it is predicted that there will be about 75 million dementia patients in the world by 2030 (Katano et al., 2020). Therefore, clarifying the pathogenesis of dementia will help to develop new treatments. A large number of studies have shown that there are many associations between loss of occlusal support and cognitive dysfunction (Bruno et al., 2014; Chen et al., 2014; He et al., 2014; Pang et al., 2015). The incidence of tooth loss increases with age. And the impairment of masticatory function caused by tooth loss can lead to corresponding cognitive dysfunction diseases such as dementia and AD (Weijenberg et al., 2011; Lexomboon et al., 2012).

Several studies have shown that the loss of molars in aged rats will lead to the decline of masticatory function and subsequent learning and memory impairment. This leads to neuronal damage in brain regions related to learning and memory, resulting in the occurrence of AD and other diseases (Onozuka et al., 2000; Kubo et al., 2005, 2017; Pang et al., 2020). However, the effect of early tooth loss on hippocampal morphology remains unknown. If the elderly is understood to suffer from AD due to tooth loss, it remains to be seen to what extent tooth loss affects the younger population. This is of great significance for the prevention and treatment of cognitive dysfunction-related diseases.

Therefore, this study hypothesized that tooth loss in youth will lead to hippocampal injury and eventually to cognitive dysfunction in juvenile rats. The right maxillary molars of 6-week-old Sprague–Dawley (SD) rats were extracted. Three months after tooth extraction, the Morris Water Maze test was used to detect learning and memory behavior, and real-time polymerase chain reaction and cresol violet staining analysis, to determine whether tooth loss in juvenile rats could cause cognitive dysfunction.

## Materials and Methods

### Animals

Twenty juvenile, SD rats aged 6 weeks were randomly divided into two groups: tooth loss (TL) group and the control (C) group (*n* = 10, each group) (weight: 185–230 g). The animals were housed under standard conditions and exposed to a 12-h light-dark cycle and had *ad libitum* access to food and water. The experiment was approved by the Animal Experiment Committee of Capital Medical University. Each rat was anesthetized using a compound anesthetic (0.15 mg/kg medetomidine, 2.0 mg/kg midazolam, and 2.5 mg/kg butorphanol). All molars in the right maxillary region were removed in the TL group. No teeth were extracted in the C group. Finally, each rat was injected with buprenorphine (0.05 mg/kg) intramuscularly to alleviate suffering during surgery.

### Morris Water Maze Test

The device consists of a circular water tank (120 cm in diameter) coated with black paint. The water tank is filled with water (24 ± 1°C). The labyrinth is divided into four quadrants. In one quadrant, place a transparent escape platform (10 cm in diameter); this platform is located 1 cm below the water surface. All rats received 5-day MWM training, 3 months after the extraction. During the training, each rat was placed into the water from the four quadrants to find an escape platform within 90 s. For rats that found the escape platform within 90 s, the time (in seconds) when they first found the platform was recorded; for rats that did not find it in 90 s, the time was recorded as 90 s. Rats that did not find the platform within 90 s during the training were artificially guided. They find the escape platform and stay on the platform for 20 s. After 5 days of training, a formal test was conducted. During the formal test, the escape platform was taken away. Each rat was placed into the water at a position opposite the platform, and they found the platform within 90 s. The time of the rats first passing the platform and the frequency of passing the platform were recorded. A CCD video camera took the test video by linking to a computer system.

### Hippocampus Collection

The rats were anesthetized with intraperitoneal injection of 3% chloral hydrate (13 mL/kg) and killed. The brain was removed and fixed in 4% paraformaldehyde for 4–6 h, and then soaked in 20% sucrose overnight. The brain tissue was cut with a cryostat (Leica, Nußloch, Germany), to create 20 μM-thick coronal sections.

### Cresol Violet Staining Analysis

After the frozen sections were washed three times with distilled water, the slides were immersed in 0.25% cresol violet (Sigma, St. Louis, MO, United States) for 30 s, washed with distilled water again, and passed through a graded series of alcohol (immersed in 70, 80, 90, and twice in 100% alcohol for 30 s each). The images were observed under an optical microscope (Zeiss, Axioskop 40, Germany) and processed using Axio vision 4.5 imaging software (Carl Zeiss).

### Immunohistochemistry

After the frozen sections were washed three times with PBS for 5 min each time, blocked with immunol staining blocking buffer (Beyotime Institute of Biotechnology, China) for 30 min, and were then incubated with the primary antibody overnight at 4°C. The sections were washed three times with PBS for 5 min each time, incubated with secondary antibodies for 1 h at room temperature, and mounted with antifade medium containing 4′,6-diamidino-2-phenylindole (DAPI). The primary antibodies used in this study were as follows: glial fibrillary acidic protein (GFAP) (1:1000, Millipore, cat #MAB360). Goat anti rabbit IgG (1:100, Alexa Fluor^®^ 488, ab150077) was used as the secondary antibody. Images of the sections were captured using a fluorescence microscope (Olympus/BX51, Japan).

### Western Blot Analysis

Proteins were extracted with Applygen total protein extraction kit. Protein concentration was normalized using Coomassie brilliant blue G-250 staining. Equal amounts of proteins were separated by SDS-PAGE on 10% polyacrylamide gel, and the proteins were transferred to PVDF membrane. After blocking with 0.1% TBST containing 5% non-fat milk at room temperature for 2 h. The membranes were blocked with 5% non-fat milk at room temperature for 1 h and were then incubated overnight at 4°C with primary antibodies, including rabbit Anti-BDNF antibody (1:1000, Abcam ab108319), rabbit Anti-AKT1 + AKT2 + AKT3 antibody (1:1000, Abcam ab179463), rabbit Anti-PAX6 antibody (1:1000, Abcam, ab195045). Rabbit Anti-GAPDH (1:1000, Abcam ab128915) was used as an internal reference. The membranes were then incubated for 20 min at 37°C with goat anti-rabbit IgG (TDY bio, S004, China) and goat anti-mouse IgG (TDY bio, S001, China) for 1 h at room temperature. The images of the bands were captured using Tanon Image software (version 4100, Shanghai, China). The color was developed using the ECL kit. Images were acquired using the Fuji Digital Science Imager and analyzed with Gel-Pro Analyzer (Version 4.0) to measure the integrated optimal density (IOD) values of specific bands. The blots were analyzed by ImageJ software (Version 1.52t).

### Real-Time Polymerase Chain Reaction

Rats were sacrificed, and brain tissue was isolated from the hippocampus on ice. Then, the hippocampi were frozen in liquid nitrogen and stored at −80°C. According to the manufacturer’s instructions, total RNA was extracted with RNAiso Plus (Takara, Japan). After the initial denaturation step at 95°C for 5 min, 40 cycles of denaturation at 95°C for 5 s, annealing at 60°C for 10 s, and extension for 30 s were carried out. Rotor gene SYBR Green RT-PCR kit (Qiagen, Japan) with Corbett rotor gene RG-3000a real-time PCR system (Rotor-Gene 6.1.93, Corbett) was used. The data were evaluated using the RG-3000a software program (rotor gene version 6.1.93, Corbett). All RT-PCR experiments were repeated thrice. The primer sequences used were as follows: rat Brain-derived neurotrophic factor (*BDNF*): 5′-CCATAAGGACGCGGACTTGT-3′ and 5′-GAGGCTCCAAAGGCACTTGA-3′; rat Tyrosine Kinase receptor B (*TrkB*): 5′-ATTGACCCAGAGAACATCAC-3′ and 5′-CAGGAAATGGTCACAGACTT-3′; rat Protein kinase B-1 (*AKT1*): 5′-GTGGCAAGATGTGTATGAG-3′ and 5′-CTGGCTGAGTAGGAGAAC-3′; rat N-methyl-D-aspartate receptor 2B (NR2B): CGCATCTGTCCACCATT-3′ and 5′-GCATCAGGAAAGCCTCG-3′.

### Statistical Analyses

SPSS 23.0 (IBM, Armonk, NY, United States) was used for data analyses. All experiments were repeated independently thrice. Data are expressed as mean ± standard deviation. The experimental data of each group showed normal distribution and homogeneity of variance. One-way analysis of variance with *t*-test was performed. *P* < 0.05 was considered to indicate statistical significance.

## Results

### Weight Change

**FIGURE 1:**
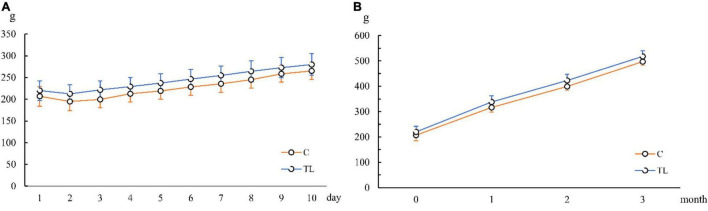
Changes of average body weight of juvenile SD rats. **(A)** Changes of average body weight in 10 days after tooth extraction. **(B)** Changes of average body weight in 3 months after tooth extraction. The orange line represents the C group and the blue line represents the TL group.

As shown in Figure 1, the body weight of the two groups decreased to varying degrees after the extraction. From 2 days after the extraction, the body weight of both groups recovered and gradually returned to the level before operation. After weight recovery, the weight of the two groups increased gradually in a similar trend.

### Morris Water Maze

**FIGURE 2:**
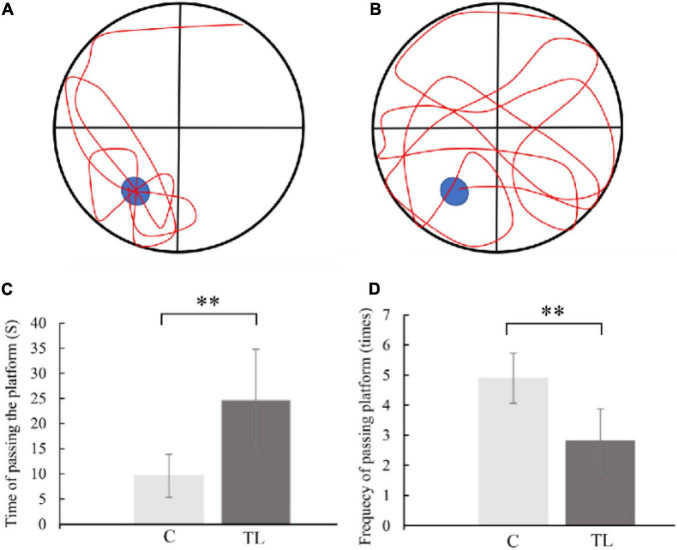
**(A)** Schematic diagram of C group looking for platform trajectory. The blue circle represents the position of life-saving platform and red represents the trajectory of movement of rats. **(B)** Schematic diagram of TL group looking for platform trajectory. The blue circle represents the position of life-saving platform and red represents the trajectory of movement of rats. **(C)** The data are expressed as the mean ± SD, ^**^indicates that there is a statistical difference between the two groups (*t*-test, ^**^P < 0.01). **(D)** The data are expressed as the mean ± SD, ^**^indicates that there is a statistical difference between the two groups (*t*-test, ^**^P < 0.01).

As shown in Figure 2, the MWM results showed that the C group performed better in finding the escape platform and had lower crossing times than the TL group (*P* < 0.01). In the C group, the average time of crossing the platform for the first time was 9.6 ± 4.27 s, and the average crossing times for the platform was 4.9 ± 0.83, and the motion trajectory was regular. The average first crossing time of the TL group was 24.6 ± 10.16 s, and the average crossing time was 2.8 ± 1.08; the motion trajectory was disordered and irregular.

### Pyramidal Cell

**FIGURE 3:**
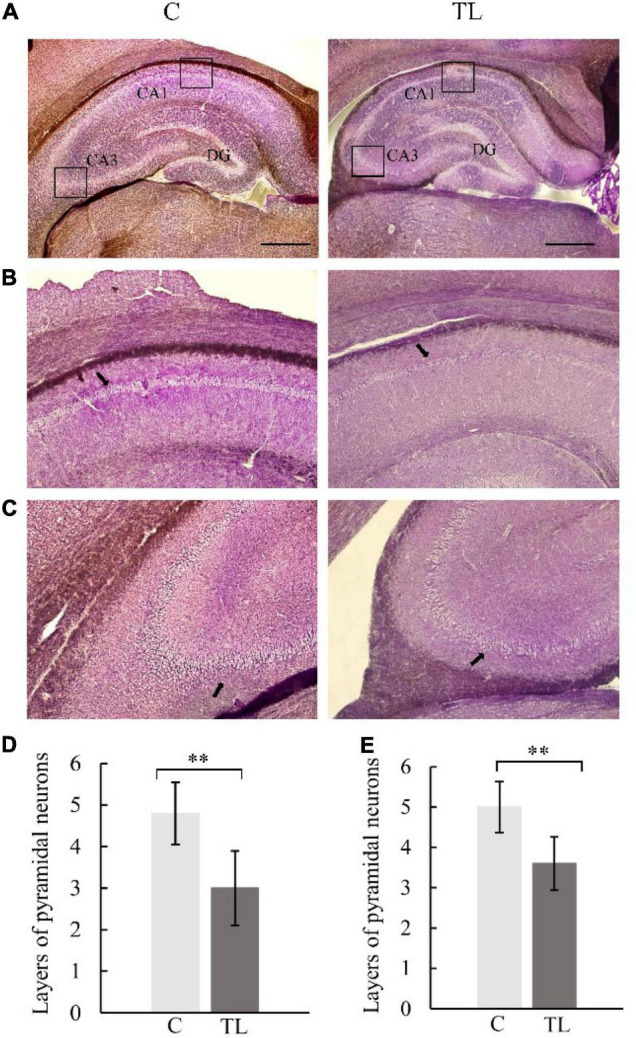
Optical micrograph of rat hippocampus. **(A)** The morphology of hippocampus in the C group and TL group was similar, with a scale of 1 mm. **(B)** Vertebral cell layer in CA1 region of the C and TL groups, with black arrow identifying the pyramidal cells. **(C)** Vertebral cell layer in the CA3 region of the C and TL groups, with black arrow identifying the pyramidal cells. **(D)** The layers number of pyramidal neurons in CA1 region in two groups. The data are expressed as the mean ± SD, **indicates that there is a statistical difference between the two groups (*t*-test, **P < 0.01). **(E)** The layers number of pyramidal neurons in CA3 region in two groups. The data are expressed as the mean ± SD, ^**^indicates that there is a statistical difference between the two groups (*t*-test, ^**^P < 0.01).

Pyramidal cell layers of different thickness were seen in hippocampal CA1 and CA3 regions of SD rats in each group (Figure 3). Further, layers of pyramidal neurons could be seen in the CA1 region were 4.8 ± 0.75 in C group, 3.0 ± 0.89 in TL group which was significantly less than C group (*P* < 0.01) and the cells were arranged in a disorderly manner. Layers of pyramidal neurons in the CA3 region were 5.0 ± 0.63 in C group, 3.6 ± 0.66 in TL group which was significantly less than C group (*P* < 0.01).

### Astrocyte

**FIGURE 4:**
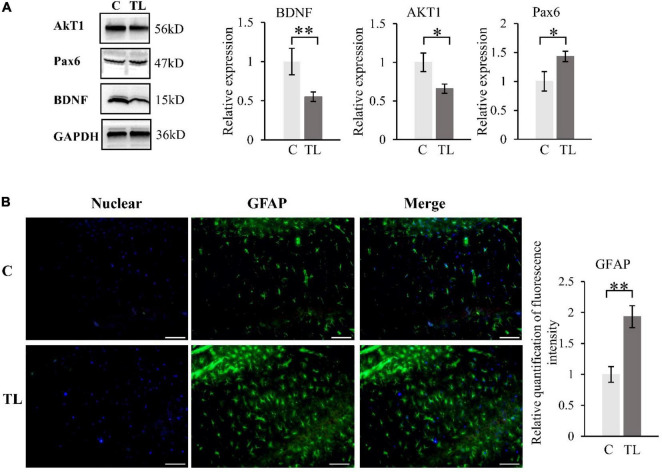
**(A)** WB results about *BDNF, AKT1* and *PAX6*. The data are expressed as the mean ± SD, *indicates that there is a statistical difference between the two groups (*t*-test, *P < 0.05, ^**^P < 0.01). **(B)** The activation of astrocytes in the CA1 of hippocampus. A Fluorescence detection of GFAP (green), Nuclear was labelled with DAPI (shown in blue). Quantification of fluorescence intensity of Astrocyte in two groups (*t*-test, **P < 0.01). The scale bar is 50 μm.

The activation of astrocytes in the CA1 of hippocampus (Figure 4B). A fluorescence detection of glial fibrillary acidic protein [GFAP (green)], a marker of activated astrocytes in hippocampus. The immunohistochemistry result showed that the number of GFAP-positive cells in CA1 of hippocampus was significantly higher in TL group than in C group.

### Western Blot Analysis

We measured and analyzed the expression of *BDNF*, *AKT1*, and *Pax6* by WB (Figure 4A). Compared to the C group, the *BDNF* and *AKT1* in the TL group decreased, while the *Pax6* increased (*t*-test, *P < 0.05, ^**^P < 0.01).

### Real-Time Polymerase Chain Reaction Analysis

**FIGURE 5:**
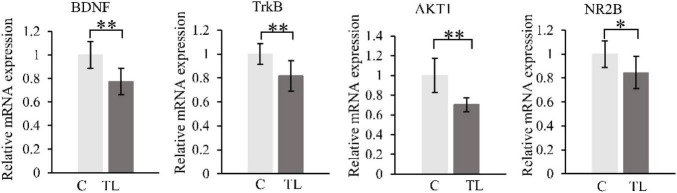
RT-PCR results. *BDNF* **(A)**, *TrkB* **(B)**, *AKT1* **(C)**, and *NR2B* **(D)**. The data are expressed as the mean ± SD, *indicates that there is a statistical difference between the two groups (*t*-test, *P < 0.05, ^**^P < 0.01).

We measured and analyzed the expression of *BDNF, TrkB, AKT1*, and *NR2B* by RT-PCR (Figure 5). Compared with the C group, the expression of *BDNF, TrkB, AKT1*, and *NR2B* in the TL group decreased by varying degrees (*P* < 0.05). *t*-test, *P < 0.05, ^**^P < 0.01.

## Discussion

Several studies have shown that the loss of teeth in elderly rats will lead to the decline of masticatory function, learning and memory impairment, and then lead to occurrence of AD and other diseases (Luo et al., 2019; Goto et al., 2020). However, whether early tooth loss in juvenile rats can affect cognitive function is still unknown. The purpose of this study was to observe the changes of hippocampal structure caused by early tooth loss in juvenile SD rats, and the subsequent impact regarding cognitive dysfunction.

In this study, we first observed that the changes of daily food intake and body weight in the C and TL groups showed a similar trend: 1 day after the extraction, food intake decreased and body weight decreased; 2 days after the extraction, the body weight gradually increased and returned to the level before tooth extraction. Therefore, in the entire study, there was no significant difference in body weight between the two groups, excluding the interference of malnutrition on this experiment (Jiang et al., 2011).

The hippocampus is crucial for memory storage and conversion (Mujawar et al., 2021; Prince et al., 2021). It contains the CA1–4 region, which is mainly composed of pyramidal neurons. The CA1 region is responsible for memory information coding, and the CA3 region is responsible for spatial information coding (Aggleton, 2012; Kesner, 2013). This study found that teeth loss in juvenile rats can lead to the decrease of pyramidal cells in CA1 and CA3 regions and the decline of hippocampal ability to recognize familiar objects and read spatial information, i.e., the decline of spatial and learning and memory ability. This is consistent with the MWM experimental results. The C group could quickly and accurately find the corresponding platform position in the MWM test after 5 days of learning. The TL group showed chaotic trajectory and unclear direction. This result indicated that tooth loss could reduce the density of neurons in CA1 and CA3 regions, and the distribution of pyramidal neurons was irregular. The results shown that tooth loss in the early age could lead to the remodeling of the ultrastructure of the hippocampus and impaired memory storage, which is manifested as a decline in learning and memory ability. Astrocytes, a type of glial cells in the brain, support essential functions such as maintenance of neurotransmitter pools, trophic support, metabolism, synaptic formation and plasticity, myelin sheath formation, injury healing, and immune surveillance (Burda et al., 2016; Manninen et al., 2020). After exogenous chemicals or trauma to the central nervous system, astrocytes in the injured area can form a glial “scar” through proliferation. This proliferation is often accompanied by increased expression of GFAP (Onozuka et al., 2000). Reactive astrocytes release of numerous effector molecules such as cytokines, chemokines, and proteases. When the brain exhibits a pathological response, reactive astrocytes induce the release of a variety of multiple harmful factors, such as *IL-1β*, *IL-6*, and *IL-17* (Araki et al., 2020). The release of these factors inhibits *BDNF* expression, reduces or cause synaptic dysfunction, impairs nerve growth and induces neuronal cell death (Cohen and Torres, 2019). We used GFAP immunostaining to determine reactive astrocyte numbers to estimate the effect of tooth loss conditions on the hippocampus. We found that the number of GFAP-positive astrocytes was significantly increased in the CA1 region in the TL group, suggesting that this region is particularly susceptible to Cognitive dysfunction due to decreased chewing ability, which is also consistent with the results in the MWM.

*BDNF* is a member of the neurotrophic factor family, which is widely distributed in the central nervous system, peripheral nervous system, endocrine system, and other regions. The *BDNF* concentration is the highest in the hippocampus (Canivet et al., 2015). It plays a role by binding with TrkB. The intracellular region of *TrkB* has intrinsic tyrosine kinase activity. After binding with *TrkB, BDNF* activates the intracellular region, resulting in enhanced self-phosphorylation of TrkB, which leads to promote the survival of nerve cells and increase synaptic plasticity and neurogenesis (Xiang et al., 2014; Peng et al., 2020; Liu et al., 2021; Wang et al., 2022). Increasing the synaptic plasticity can affect long-term potentiation and enhance the learning process and memory formation. This study shows that tooth loss reduces the expression levels of *BDNF* and *TrkB*, resulting in the weakening of learning ability and long-term memory ability of juvenile SD rats. However, *BDNF* can activate phosphatidylinositol 3-kinase (*PI3K*)-protein kinase B (*Akt*) pathway after binding to the *TrkB* receptor (Jeong et al., 2021; Li et al., 2021). This pathway can regulate the proliferation and differentiation of neural stem cells and repair neuronal injury (Lv et al., 2016; Fu et al., 2020). This study shows that tooth loss reduces the expression level of *AKT1*, thereby inhibiting the proliferation and differentiation of neural stem cells and the repair of neuronal damage, which also leads to the weakening of learning ability and long-term memory ability of juvenile SD rats. The same results also appeared in the expression of *NR2B. NR2B* has an important relationship of N-methyl-D-aspartic acid (NMDA) nerve endings in the brain, which is related to memory function (Faure et al., 2007; Melike et al., 2021). This study also showed that tooth loss reduces the expression of *NR2B*, and then reduces the learning ability of juvenile SD rats. *Pax6* is an important transcription factor for eye, brain and olfactory system development, some researchers found that there are probably more targets and downstream pathways of *Pax6* involved in Alzheimer’s disease pathogenesis (Dunckley et al., 2006; Zhang et al., 2021). And *Pax6* expression is increased in the brains of APP transgenic mice and human Alzheimer’s disease patients. In our research, it was also found that tooth loss leads to increased expression of *Pax6*, which leads to cognitive impairment in rats.

We speculate that after tooth loss in juvenile SD rats, dental pulp- and periodontal membrane-derived neural signals are reduced, and afferent nerve fibers in the trigeminal nucleus are reduced. On the one hand, it affects *BDNF/TrkB* and *PI3K/Akt* pathways to inhibit the proliferation and differentiation of neural stem cells and the repair of neuronal damage. On the other hand, increasing the expression of *Pax6* increased the amyloid-β-induced neuronal death and tau phosphorylation, eventually lead to the occurrence of AD and other diseases.

The limitation of this study is that we extracted all the right maxillary molars of juvenile SD rats, and set up different experimental groups for the number of lost teeth. Therefore, in future studies, we hope to explore whether the number of lost teeth has an impact on the learning and memory ability of juvenile SD rats.

## Conclusion

Tooth loss in juvenile SD rats will reduce synaptic function, impairs nerve growth and induces neuronal cell death in the hippocampus, and inhibit the proliferation and differentiation of neural stem cells and the repair of neuronal damage, finally lead to cognitive dysfunction.

## Data Availability Statement

The raw data supporting the conclusions of this article will be made available by the authors, and the generated datasets are available by request to the corresponding author.

## Ethics Statement

The animal study was reviewed and approved by the Animal Experiment Committee of Capital Medical University.

## Author Contributions

JH contributed to the design of the experiments, collection and assembly of all data, data analysis and interpretation, and manuscript writing. XW contributed to the design of the experiments and collection and assembly of all data. WK participated in the data collection and analysis. QJ conceived the idea and contributed to data interpretation. All authors have approved the final version of the manuscript.

## Conflict of Interest

The authors declare that the research was conducted in the absence of any commercial or financial relationships that could be construed as a potential conflict of interest.

## Publisher’s Note

All claims expressed in this article are solely those of the authors and do not necessarily represent those of their affiliated organizations, or those of the publisher, the editors and the reviewers. Any product that may be evaluated in this article, or claim that may be made by its manufacturer, is not guaranteed or endorsed by the publisher.
